# Recapitulating the Micromechanical Behavior of Tension and Shear in a Biomimetic Hydrogel for Controlling Tenocyte Response

**DOI:** 10.1002/adhm.201601095

**Published:** 2016-12-27

**Authors:** Dharmesh Patel, Sadhana Sharma, Stephanie J. Bryant, Hazel R. C. Screen

**Affiliations:** ^1^ School of Engineering and Materials Science Queen Mary University of London Mile End Road London E1 4NS UK; ^2^ Department of Chemical and Biological Engineering University of Colorado Boulder Boulder CO 80303 USA; ^3^ Department of Chemical and Biological Engineering Material Science and Engineering Program BioFrontiers Institute University of Colorado Boulder CO 80303 USA

**Keywords:** fiber composites, mechanical properties, mechanotransduction, poly(ethylene glycol), tenocyte

## Abstract

**A fiber composite system** is presented which recapitulates the fiber‐composite‐like nature of tissues and generates similar modes of shear and tension. The shear/tension ratio can be customized during composite manufacture and incorporates viable cells. The system is a valuable tool for mechanotransduction research, providing a platform with physiologically relevant conditions for investigating cell behavior in different tissue types.

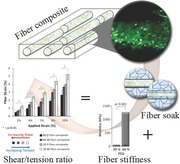

Nearly all connective tissues within the body are fiber composites of some nature, composed of fibers made of collagen or elastin, embedded in a soft proteoglycan‐rich matrix. Tendons and ligaments provide some of the simplest examples of this arrangement, where the fibers are almost entirely collagen, hierarchically organized, and highly aligned to efficiently transmit unidirectional forces.^1^, ^2^ When fiber composite materials are loaded during normal physiological use, the constituent fibers will both stretch and slide past one another in response to the applied strain. The fiber composite nature of tendon has received some interest. In tendon, the cells (tenocytes) are arranged in rows along the fibers,^3^, ^4^ where they will experience tension and shear when tendon is loaded and in ratios that are similar to those seen at the fiber level as the fibers stretch and shear. The complex, fiber composite nature of many tissues found in the body results in complex, multimodal strain distributions at the cell level. Recapitulating these complex strain profiles is important to understanding physiological and pathological strains that are experienced by cells.

In vitro models offer important biological tools to investigate cell behavior under specific, controllable, and reproducible conditions, overcoming confounding factors found in in vivo systems such as compositional and structural variations within tissue. Such models are particularly important during the investigation of mechanical cues, where knowledge of the strain input is necessary to confidently ascertain the effects of mechanical stimuli. In vitro model systems typically study the effects of mechanical cues on cells when they are homogenously seeded onto 2D or within 3D materials.^5^, ^6^, ^7^, ^8^, ^9^, ^10^ When combined with bioreactors, excellent control over external levels of tensile or compressive strains is achieved. However, the local strains that are transmitted to the cells depend highly on the material. For isotropic materials (e.g., cells encapsulated in a bulk 3D hydrogel), strains are generally transferred homogeneously to the cells, achieving a single deformation mode.^11^, ^12^ Conversely, for anisotropic materials, (e.g., cells seeded into porous or fibrous scaffolds) strain transfer is highly heterogeneous and difficult to predict. Systems that investigate cell response to shear generally utilize fluid‐induced shear stress,^13^, ^14^ but shear is then created in the absence of strain. Fluid‐induced shear stress has also been combined with direct mechanical stretch, trying to mimic the local cell environment of fiber composites, combining shear with tension.^15^ However, none of the current in vitro models are able to recapitulate the complex micromechanical environment of fibrous tissues with simultaneous shear strain created from fiber sliding and tensile strain created from fiber stretch. Current systems, therefore, lack the ability to study cellular response to a combined shear and tension strain environment.

In this work, our goal was to develop a cell‐based in vitro model that recreates the combined shear and tension local strain environments found in biological tissues, herein recreating those strains found in tendon and ligaments. The ability to control the local strain environment applied to cells and create multimode strain conditions is crucial to understanding the effects of mechanical cues on cells under physiological and pathological conditions. First, we characterized the local micromechanics in healthy native tendon and then used this knowledge to design a biomimetic hydrogel architecture that captures multimode strain conditions.

Tendon is 60%–90% collagen by dry weight,^16^, ^17^ arranged in a hierarchical manner in the primary loading direction (**Figure** **1**A). Proteoglycan‐rich matrix between the collagen units throughout the tendon hierarchy modulates the extent of collagen sliding and shear at each hierarchical level.^1^, ^18^, ^19^, ^20^, ^21^, ^22^, ^23^, ^24^ The extension mechanisms at different levels of the tendon hierarchy have been widely studied,^25^, ^26^, ^27^, ^28^ and fiber extension seen to be ≈40% of that applied to the whole tissue.^29^ The cells within tendon are arranged in rows attached to the collagen fibers (Figure 1A, inset), experiencing a combination of shear and tension as the tendon is loaded. By using the cells as local strain markers, or tracking the collagen directly, the local strains along fibers were quantified from microscopy images as different tendon explants were strained (Figure 1B,C). Using this approach, local cell strains were estimated for different amounts of applied strain in different tendon types. Here, local fiber and cell strains were measured at a physiologically relevant whole tendon strain of 5% and ranged from 1% to 2.6%, or 21% to 52% of the applied values (Figure 1C).

**Figure 1:**
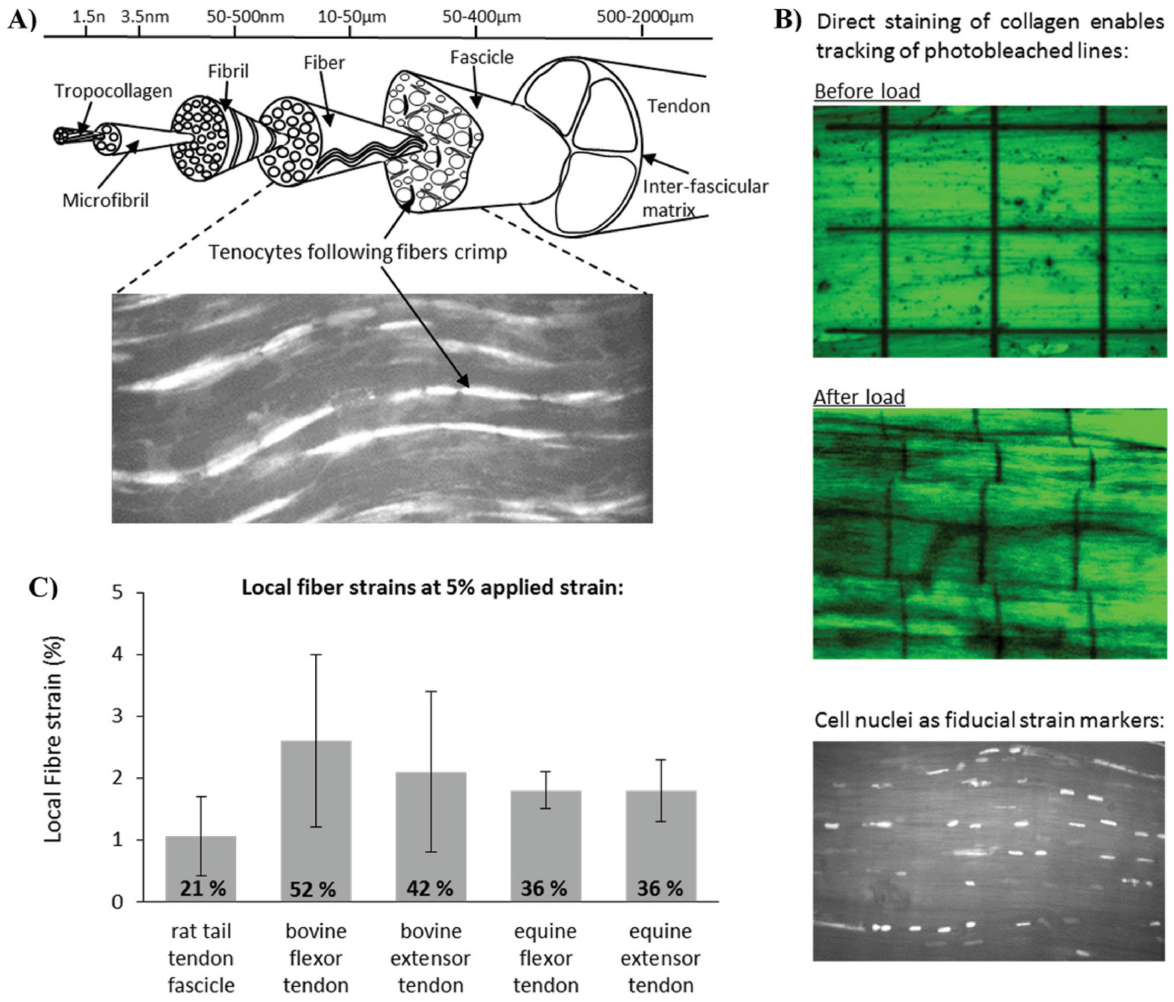
A) Schematic depicting the hierarchical structure of tendon, in which collagen is aligned parallel to the loading axis. Cells (tenocytes) are arranged in rows along the fibers and can be visualized with confocal microscopy (see the inset). B) Adopting a range of different staining technologies, it is possible to visualize the local strains in tendon fibers when the whole tissue is subject to applied strain. Stained samples were secured in a custom designed loading rig, and subjected in increasing applied strain, recording the extent of fiber strain and fiber sliding in response to applied strain. Data across a series of different tendon types highlight that local strains along fibers are significantly smaller than applied strains. C) At 5% applied strain, local fiber strains ranged from 1%–2.6%, or 21%–52% of the applied values.

A 3D hydrogel model system was developed to capture this combined phenomena of fiber extension and fiber sliding that is observed in native tendon, recapitulated as local fiber tension and fiber shear. This 3D model is fabricated from a single cytocompatible chemistry based on cross‐linked poly(ethylene glycol) (PEG) made from PEG dimethacrylate (PEGDM) macromolecular monomers, but with an architecture that is designed to transfer the externally applied strains into highly controllable local tension and shear strains. The manufacturing procedure and the resulting architecture is schematically depicted in **Figure** **2**. The model is a fiber composite material that consists of discontinuous hydrogel fibers embedded in a bulk hydrogel matrix. The degree of interconnection between the fibers and the bulk matrix, termed the fiber–matrix integration, can readily be tuned by controlling diffusion of precursors into the fibers prior to polymerization of the bulk hydrogel matrix and encapsulation of the fibers, thus creating a semi‐interpenetrating network at the interfaces.^30^ The synthetic chemistry offers further tunability with respect to the mechanical properties of the fibers and independently of the bulk matrix. Finally, biological moieties (e.g., cell adhesion peptides) are readily introduced into the fibers during hydrogel formation enabling localized cell attachment to the fibers, similar to that observed in tendons and ligaments.

**Figure 2:**
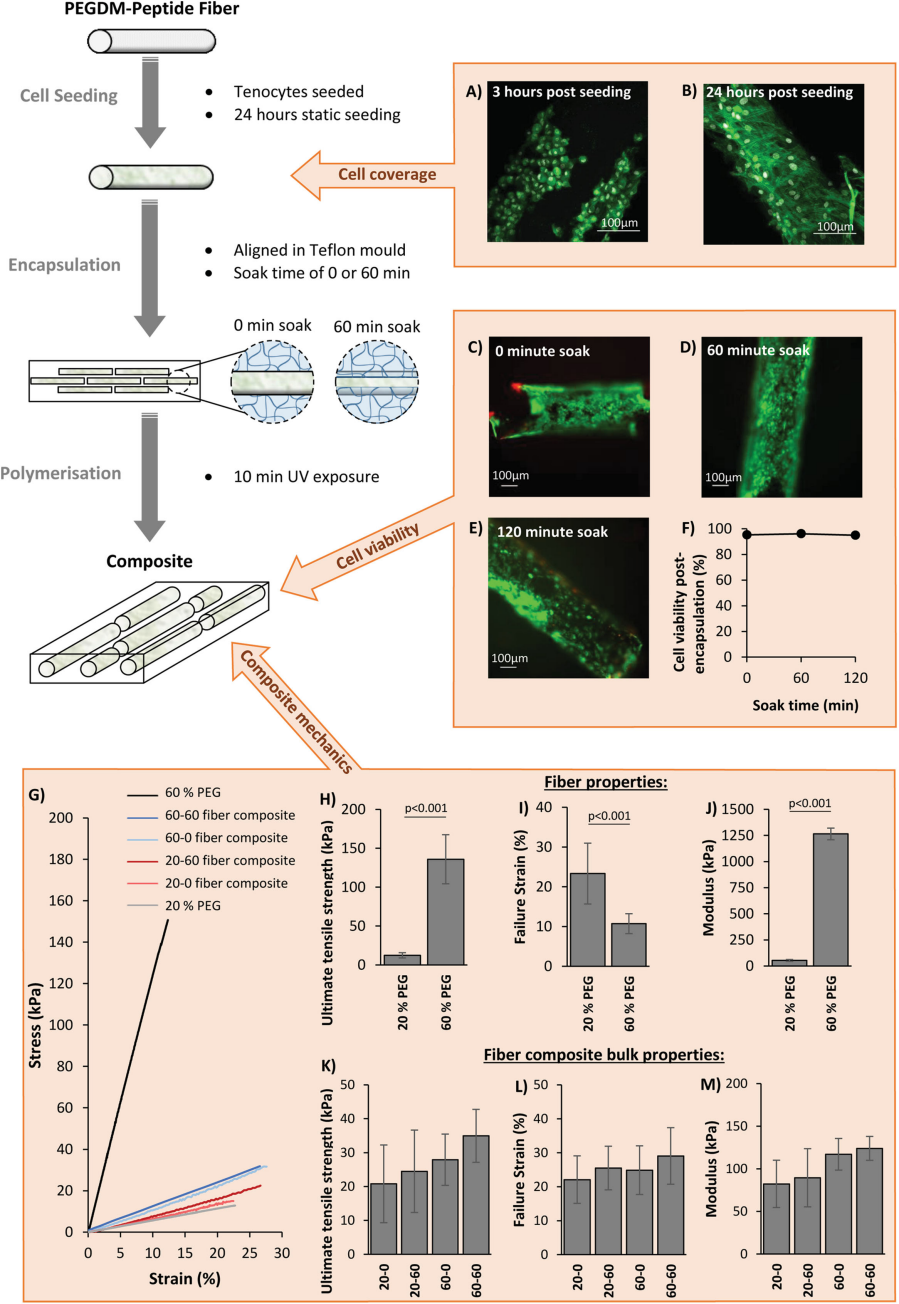
Composites are manufactured via a three‐step procedure as shown in the schematic on the left. Peptide‐PEG fibers are first seeded with cells in a static culture condition for 24 h, after which they are encapsulated in a hydrogel precursor solution with PEGDM and photoinitiator. The solution is then left to soak for a fixed amount of time to control the extent of precursor penetration into the fibers, and then polymerized under ultraviolet light to produce the final composites. Confocal images of tenocytes (stained with Alexa Fluor 488 Phalloidin (green) and 4′,6‐diamidino‐2‐phenylindole (DAPI) (gray) to show the F‐actin in the cytoskeleton and the nucleus, respectively) after 3 h A) postseeding on PEG‐YRGDS fibers showed cells attach to fibers, but exhibit a compact spherical shape. B) After 24 h the cells are spreading across the fiber surface and possessing longer F‐actin filaments. C–F) Soaking cell seeded fibers for either 0, 60, or 120 min in 20% PEG matrix solution before polymerization did not significantly affect cell viability (images C–E show cells stained with Calcein AM (green) and ethidium homodimer (red), and graph F shows average cell viability after 0, 60, or 120 min soak before polymerization, *n* = 6). 60% PEG was found to be significantly stiffer than 20% PEG, showing a higher ultimate tensile strength (H), failure strain (I), and modulus (J) than 20% PEG. Average bulk mechanical properties of composites made with 20% or 60% PEG‐YRGDS fibers seeded with cells and soaked for either 0 or 60 min prior to polymerization (*n* = 9–13) were found to be similar between composite types, with no significant difference between ultimate tensile strength (K), failure strain (L), and modulus (M). Bar graphs show mean with SD as error bars and composite types are labeled as “XX–YY” where “XX” describes the % PEGDM used to make fibers, and “YY” is the fiber soak time in minutes prior to polymerization. “*p*” values represent significant differences between groups as calculated from a one way ANOVA and Tukey comparison of means.

Here, the cell adhesion peptide tyrosine–arginine–glycine–aspartic acid–serine (YRGDS) was covalently attached to the fibers to demonstrate efficacy of the model. YRGDS was chosen because the arginine–glycine–aspartic acid (RGD) motif is commonly used as a cell attachment peptide in studies.^31^, ^32^, ^33^, ^34^, ^35^ It is present in many extracellular matrix proteins including fibronectin and collagen.^36^, ^37^ However, any peptide can be incorporated allowing the fiber composite system to be tailored toward different tissue types. Tenocytes readily attached to the PEG‐YRGDS fibers within three hours of seeding, exhibiting a circular morphology (Figure 2A). At 24 h postseeding, cell spreading across the surface of the fibers was observed, with long F‐actin cytoskeleton arrangements (Figure 2B), indicating good attachment with cell coverage estimated to be ≈68% of the fiber surface area. High viability was also confirmed during the encapsulation process and was maintained when fiber soak times of up to two hours were adopted. The soak time controls the interfacial characteristics between fiber and bulk matrix. While cells are exposed to macromolecular monomers and photoinitiator molecules during the soak time, the cells do not appear to be negatively affected. This result is consistent with other reports which have shown that for PEGDM molecules of sufficiently high molecular weight and low concentration, similar to that used here, viability is not affected over a similar time frame.^38^ Similarly, other reports have confirmed that cells exposed to photoinitiator molecules and subsequently irradiated experience minimal radical‐induced damage.^39^, ^40^ Overall, the manufacturing process maintains tenocyte viability.

The tunability of the fiber composite system was investigated, assessing the effects of fiber stiffness and fiber soak time on both the bulk mechanical properties of cell‐laden fiber composites and their local mechanics. The fiber stiffness was controlled by varying the percent PEGDM from 20% to 60% in the precursor solution prior to fiber formation. Fibers prepared from 60% PEGDM were significantly stiffer than those prepared from 20% PEGDM (1300 ± 60 kPa and 53 ± 9 kPa, respectively) and also had a higher ultimate tensile strength (140 ± 30 kPa and 12 ± 3 kPa, respectively) (Figure 2G–J). Using these two degrees of fiber stiffness, four different cell‐laden fiber composite materials were manufactured, using a bulk hydrogel matrix manufactured from 20% PEGDM in all composites and either a 0 or 60 min fiber soak time. The bulk mechanics of all four fiber composites were similar and mirrored the properties of the bulk 20% PEGDM hydrogel without fibers (Figure 2G–M). No significant differences were observed between the failure strength, failure strain, or modulus of any of the composite types; although increasing fiber soak time showed an increasing trend for each of these parameter, while increasing fiber stiffness led to a trend for increased composite stiffness. Such similarities in gross mechanics are expected given the low volume of fibers (≈8%) in the composites.

In contrast to the bulk mechanical data, manipulating fiber stiffness and/or soak time led to significant differences in local micromechanics across the four different fiber composites (**Figure** **3**A,B). Each fiber composite type was subjected to gross applied strains of 2%, 4%, 5%, 8%, and 10% and fiber extension was monitored by brightfield microscopy at ×10 magnification (Figure 3A). As anticipated from fiber composite theory, stiff fibers (60% PEG) stretched less within composites across all gross strains that were investigated, while increasing fiber soak time from 0 to 60 min, increases fiber–matrix integration, and resulted in more transfer of the applied strain to the fibers to increase fiber extension.

**Figure 3:**
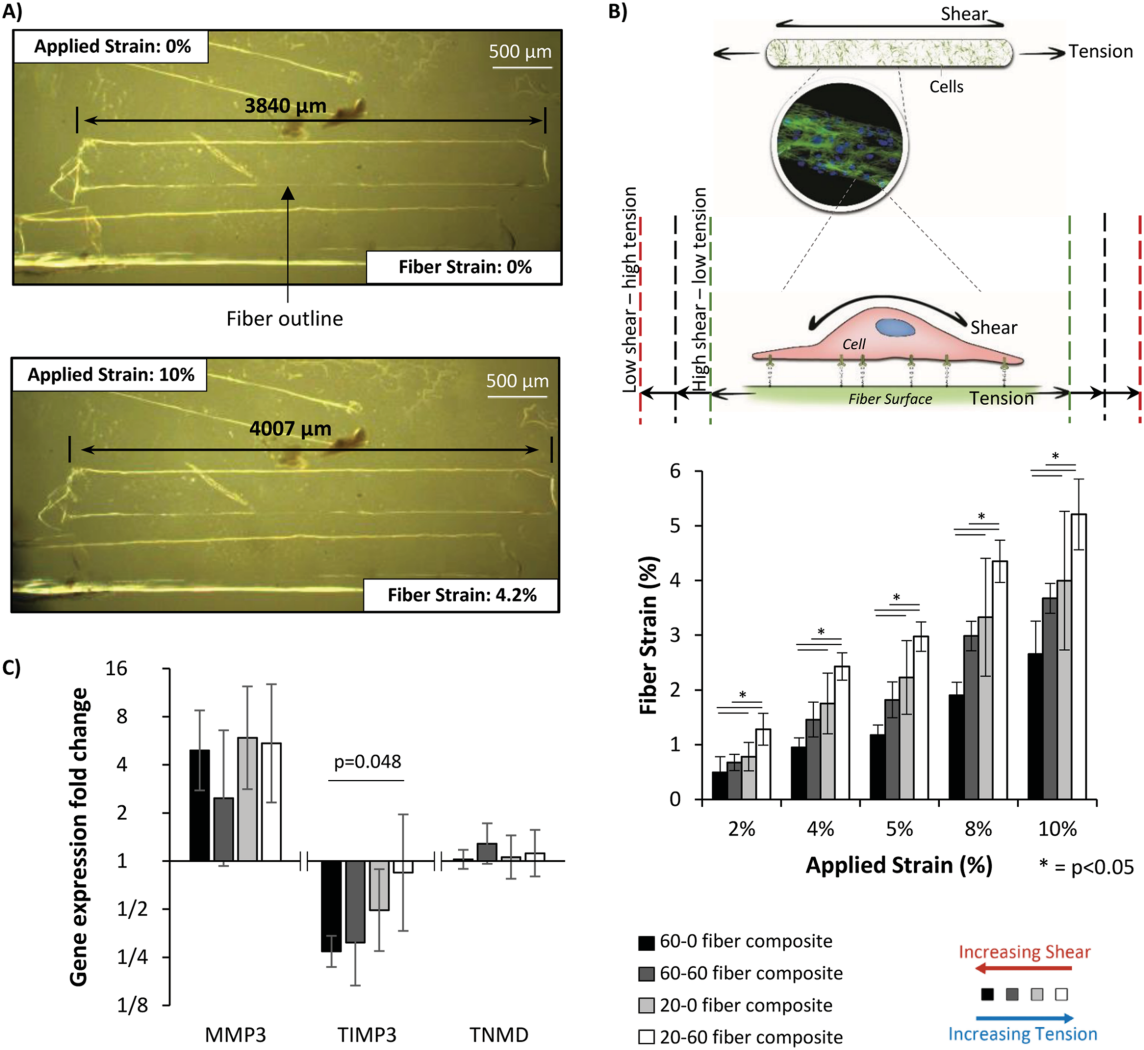
A) Example of fiber strain analysis used to characterize local micromechanics of the composites. Brightfield images of composites were taken at different levels of applied strain and the fibers within identified by the difference in refractive index along the fiber edge, leading to an apparent white outline. The length of the fiber was measured as indicated by black arrows. B) Local strain along a fiber could be altered by using either different soak times or degrees of fiber stiffness during composite manufacture (*n* = 8–15). This includes the physiological conditions found in tendons (40% fiber tension) or nonphysiological conditions such as low shear–high tension (≈60% fiber tension generated by manufacturing composites with 20% PEG fibers and a 60 min soak time) or high shear–low tension (≈24% fiber tension and generated by manufacturing composites with 60% PEG fibers and a 0 min soak time). C) Using these composites, gene expression of matrix metalloproteinase‐3 (MMP‐1), tissue inhibitor of metalloproteinase 3 (TIMP3), and tenomodulin (TNMD) was analyzed after 24 h of cyclic loading. Expression was calculated using the Pfaffel efficiency corrected method^44^ with nonstrained samples used as relative controls and normalized to reference gene L30.^34^, ^40^ Bars indicate mean expression fold change and error bars indicate standard deviation (*n* = 3). Graphs show mean ± SD. *P* values are results from post‐hoc Tukey HSD tests.

With the degrees of fiber stiffness and soak time adopted in this study, a range of shear/tension ratios is producible by the system. For example, at 5% applied strain, the fibers within composites made with 60% PEG fibers and no soak time stretched 1.2%, while the fibers in composites made with 20% PEG fibers and a 60 min soak stretched 3% (Figure 3B). While difficult to determine the precise degree of shear on the cells in each composite type, fiber composite theory tells us that the less fibers within the composite extend, the greater the shear will be along the fiber surface.

The range of physiological fiber strains found in functionally distinct tendons, 21%–52% of the applied strain (Figure 1C), can be recreated by using either 20% PEG fibers with a 0 or 60 min soak time, or 60% PEG fibers with a 60 min soak time. Furthermore, it is possible to produce other ratios for different applications, such as a higher tension condition (60% fiber tension), produced using 60% PEG fibers with a 0 min soak time. Additional structural characterization showed the fiber diameter (≈300 µm) and fiber‐to‐fiber distance (≈400–600 µm) to be considerably larger than that of tendons. However, the aim of this system is to recreate the local strain environment of tendon tissue, thus capturing shear and tension at the cellular level.

Tenocyte strain response within the fiber composite system was analyzed through investigating gene expression of some key markers of tendon health and disease; matrix metalloproteinase‐3 (MMP‐3), which cleaves various matrix proteins and is found to decrease in tendinopathic tissue;^41^, ^42^ tissue inhibitor of metalloproteinases‐3 (TIMP‐3), which inhibits many matrix degrading enzymes and is decreased in tendinopathy;^42^ and tenomodulin (TNMD), a transmembrane glycoprotein associated with the tendon phenotype.^43^ Previous time‐course analyses of gene expression in tenocytes have indicated that COL‐1 gene expression peaks around 18–24 h after the start of the loading period while MMP‐1 downregulation peaks around 48 h.^10^ Consequently, a 24 h loading period, a common loading period for tenocyte studies,^10^ and the strain conditions classically used in the large majority of tenocyte mechanotransduction studies (5% cyclic strain at 1 Hz) were chosen as appropriate parameters for assessing gene expression changes as a result of different shear/tension ratios. The resulting gene expression from the resident tenocytes within each composite type was then analyzed to investigate the cell response to physiological and nonphysiological local strain conditions (Figure 3C). The study found that TIMP‐3 gene expression was significantly downregulated with increased shear and reduced tension, while TNMD and MMP‐3 showed no significant changes with shear/tension ratios. Additionally, MMP‐3 gene expression was generally upregulated with loading in the majority of local strain conditions whereas TNMD gene expression was not affected by loading.

The preliminary data shown here support the idea that even small local changes in shear and tension regulate tenocyte behavior, despite the external loads being maintained. The data confirms other studies which have demonstrated that fluid shear downregulates TIMP3,^13^ but importantly it additionally highlights cell sensitivity to these cues. Further, although previous studies have implicated shear as a regulator of matrix turnover in tendons,^13^, ^14^ the magnitudes of shear applied in those studies were not physiologically relevant, and the accompanying tensile strain absent. While we do not yet know the significance of the gene expression changes instigated by the different shear/tension ratios under a constant applied external strain, the ability of tenocytes to regulate gene expression in response to these small changes demonstrates the high sensitivity of tenocytes to altered shear stimuli.

In conclusion, the novel fiber composite system developed in this study provides the first system able to recreate highly controlled combined levels of cell shear and tension. Other in vitro systems^6^, ^8^, ^13^, ^14^, ^15^ do not recapitulate the native tissue environment, resulting in studies where nonphysiological magnitudes and modes of shear or tension are applied. This is further confounded by the high mechanosensitivity exhibited by cells such as tenocytes. The fiber composite system addresses this shortcoming of current in vitro systems and is particularly suited for mechanotransduction studies in complex tissues such as tendons. The optical transparency of the system also facilitates investigations concerning cell morphology and deformation via microscopy under different strain conditions. Through alteration of parameters, such as the cell attachment peptide, fiber stiffness, or fiber soak time, the fiber composite system has the potential to be tailored toward specific strain environments. Preliminary data from applying the fiber composite system for tendon research suggests that the cellular shear/tension ratio is an important factor regulating cell behavior in tendons, and hence important in the development and progression of tendinopathies. The shear/tension ratio could potentially regulate other cell types, thus the fiber composite system opens up new possibilities for future studies where more physiologically relevant conditions can be used to elucidate cell behavior in different tissue types.

## Experimental Section

Details of all materials used in the study are provided in Table S1 in the Supporting Information.

*Characterizing Cell Strains in Tendon Explants*: Tendons (*n* = 5 minimum) were sourced from either a local abattoir (bovine: deep digital flexor tendon and common digital extensor tendon (Blixes Farm, Chelmsford, UK); equine superficial digital flexor tendon and common digital extensor tendon (Potters, Taunton, UK)) or as waste tissue from unrelated experiments (rat tail tendon fascicles (Queen Mary University of London, UK)). Individual fascicles (see Figure 1A), at least 20 mm in length, were carefully dissected from each tendon (*n* = 3 minimum), and maintained under Dulbecco's modified Eagle's medium (DMEM) hydration until use.

Bovine and rat tail fascicles were incubated in 5 × 10^−6^ m Acridine Orange in DMEM for 40 min, to stain the cell nuclei. Equine fascicles were stained with the collagen stain 5‐([4,6‐dichlorotriazin‐2‐yl]amino)fluorescein hydrochloride at a concentration of 2 mg mL^−1^ in 0.1 m sodium bicarbonate buffer, pH 9 for 20 min. Following staining, all fascicles were washed in two changes of DMEM for 20 min, prior to securing in a custom made tensile testing rig,^30^ at a resting length of 10 mm. Fascicles were maintained in DMEM for the duration of the experiment.

A tare load of ≈0.1 N (range: 0.05–0.15 N) was applied to fascicles, and the initial sample length measured to define a zero strain condition. For equine fascicles, a grid of four squares, each 50 µm × 50 µm was photobleached onto the central region of the fascicle (Figure 1B, top). In bovine and rat tail fascicles, the cell nuclei could be viewed with the confocal microscope (Figure 1B, bottom). An image of the photobleached grid or cells was taken in a focal plane ≈20–25 µm from the sample surface at the zero strain condition. Fascicles were then incrementally strained, locating either the grid or the same cells at each increment, before reimaging the sample. Deformation of the grid or movement of the cell nuclei were utilized to determine the local strains along the collagen fibers at each increment. Local fiber strains are plotted for each tendon type at an applied strain of 5% (Figure 1C).

*Cell Source*: Tenocytes were isolated from bovine extensor tendons via explant outgrowth or direct tendon digestion (1 U mL^−1^ dispase and 2 mg mL^−1^ collagenase type II for 48 h at 37 °C). In the former, tenocytes at passage four to five were used for the viability and cell attachment studies. In the latter, tenocytes were directly incorporated into the fiber composites for gene expression studies. Similar attachment and viability in the fiber composites was observed with both isolation methods. Tenocytes were cultured in tenocyte medium containing DMEM (with low glucose and pyruvate), 4‐(2‐hydroxyethyl)‐1‐piperazineethanesulfonic acid (HEPES) buffer, l‐glutamine, nonessential amino acids, penicillin/streptomycin, and fetal bovine serum (FBS) in a ratio 100:2:1:1:1:10, respectively.

*Fiber Composite Manufacture*: PEGDM was synthesized as described previously.^45^ Acrylate‐PEG‐YRGDS was synthesized by reacting 1 mol acrylate‐PEG‐NHS to 1.1 mol peptide (YRGDS) at pH 8.4. Fibers were fabricated by polymerizing a precursor solution of 20% or 60% PEGDM in phosphate buffered saline (PBS) (pH 7.4) with 0.05% w/v Irgacure 2959 and 5 × 10^−3^ m acrylate‐PEG‐YRGDS in a Teflon mold (0.3 mm diameter and 4 mm length) under UV light (365 nm, ≈4 mW cm^−2^) for 10 min. Fibers were sterilized in 70% ethanol and then rinsed with sterile PBS. Fibers were seeded with tenocytes in nontissue culture treated well plates at ≈3.5 million tenocytes per 150 fibers per well in tenocyte media—FBS for 1.5 and then in full medium overnight. Fiber composites were fabricated by positioning the cell seeded fibers into sterile rectangular Teflon molds (25 mm × 2.5 mm × 1 mm), slowly injecting a sterile filtered precursor solution of 20% PEGDM with 0.05% w/v Irgacure 2959 in tenocyte medium, and polymerizing after 0 or 60 min under UV light (365 nm, ≈4 mW cm^−2^, 10 min). Cell‐seeded fibers or full fiber composites were maintained in tenocyte medium incubated at 37 °C with 5% CO_2_.

*Tenocyte Analysis*: After prescribed times, cell seeded fibers were fixed, cell membrane permeabilized, stained with Phalloidin Alexa Fluor 488, and nuclei counterstained by 4′,6‐diamidino‐2‐phenylindole (DAPI) following standard procedures. Z‐stack images of fibers were taken at three hours and 24 h postseeding (*n* = 3) by confocal microscopy (SP2, Leica) to quantify cell attachment. Cell viability was characterized in the cell seeded fiber composites immediately after polymerization using Calcein AM (4 × 10^−6^ m) and ethidium homodimer (4 × 10^−6^ m) by imaging two locations on each fiber per soak time (*n* = 6) with an epifluorescent microscope (DMI 4000B, Leica). Cell coverage was estimated from Calcein AM stained cell seeded fibers using confocal microscopy of fibers at 10× magnification (6 fibers, 2 locations each) were taken and the surface area of fibers covered with viable cells relative to the surface area of the whole fiber calculated.

*Composite Mechanical Properties*: Composites (*n* = 9–13 for each composite type, with each composite containing seven fibers) were loaded to failure at 15% min^−1^ using the method described previously^30^ and modulus, failure strain, and ultimate tensile stress computed. Specimens made from 20% (*n* = 10) or 60% PEGDM (*n* = 11) were also tested. The micromechanics were assessed using a custom uniaxial strain rig^30^ with brightfield microscopy to image fibers within composites while applying strain (*n* = 8–15 per composite type, with seven fibers/composite). Composites were incrementally stretched up to 10% strain, at 15% strain min^−1^, with images taken at 0%, 2%, 4%, 5%, 8%, and 10% applied strain. At each applied strain, local fiber tensile strains were calculated as a percentage tension of the fiber strain as a percentage of the gross applied strain by analyzing the images in ImageJ.^46^

*Tenocyte Mechanotransduction and Gene Expression Analysis*: Composites designated as strained were placed into individual wells in a sterilized custom strain rig^30^ and in an incubator. A loading regime of 5% cyclic strain (sinusoidal waveform, 1 Hz frequency) was applied for 24 h while nonstrained control samples were kept in separate wells of a 6‐well plate in the same incubator. After loading, all composites were snap frozen in liquid nitrogen, RNA extracted via tissue homogenization, and a MiRNeasy Micro Kit and reverse transcribed using a High‐Capacity cDNA Reverse Transcription Kit with custom primers (Table S2, Supporting Information), and real‐time PCR performed. Gene expression was analyzed using the Pfaffl efficiency corrected method,^44^ normalized to the housekeeping gene L30,^34^, ^40^ and displayed as fold changes of strained samples relative to nonstrained controls. Four composites were made per treatment group; 2 strained and 2 nonstrained controls, and the experiment was performed with three biological repeats.

*Statistical Analysis*: Data were analyzed for statistical significance using a one way ANOVA and a significance level of 0.05, followed by Tukey HSD tests.

## Supporting information

As a service to our authors and readers, this journal provides supporting information supplied by the authors. Such materials are peer reviewed and may be re‐organized for online delivery, but are not copy‐edited or typeset. Technical support issues arising from supporting information (other than missing files) should be addressed to the authors.

Supplementary
