# Supplementary material for: Recapitulating the Micromechanical Behavior of Tension and Shear in a Biomimetic Hydrogel for Controlling Tenocyte Response
Source: Adv Healthc Mater. 2016 Dec 27;6(4):1601095. doi: 10.1002/adhm.201601095 (PMC5469035; doi:10.1002/adhm.201601095)
Supplement: Supplementary file 1 — Supplementary [file ADHM-6-na-s001.pdf]

Copyright WILEY-VCH Verlag GmbH & Co. KGaA, 69469 Weinheim, Germany, 2016.

ADVANCED HEALTHCARE MATERIALS

Supporting Information

for *Adv. Healthcare Mater.*, DOI: 10.1002/adhm.201601095

Recapitulating the Micromechanical Behavior of Tension and Shear in a Biomimetic Hydrogel for Controlling Tenocyte Response

*Dharmesh Patel, Sadhana Sharma, Stephanie J. Bryant, and Hazel R. C. Screen**

Supporting Information

Recapitulating the Micromechanical Behavior of Tension and Shear in a Biomimetic Hydrogel for Controlling Tenocyte Response

*Dharmesh Patel, Sadhana Sharma, Stephanie J. Bryant and Hazel R. C. Screen**

Table S1: List of all materials used within study, including abbreviations and supplier details.

Chemical or Material	Supplier	Product no.
5-([4,6-Dichlorotriazin-2-yl]amino)fluorescein hydrochloride (5-DTAF)	Sigma Aldrich, UK	D0531
Acridine Orange	Invitrogen, USA	A1301
Acrylate-PEG-NHS	JenKem Technology, USA	A5022-1
Bovine Serum Albumin (BSA)	Sigma Aldrich, UK	A3311
Calcein AM	Life Technologies, UK	C1430
Chloroform	Sigma Aldrich, UK	C2432
Collagenase Type II	Worthington Biochemical Corporation, USA	CLS-2
DAPI (4',6-Diamidino-2-Phenylindole, Dilactate) (DAPI)	Life Technologies, UK	D3571
Dichloromethane	Sigma Aldrich, UK	D65100
Diethyl Ether	VWR International Ltd, UK	23809.328
Dispase Solution, 1U/mL	STEMCELL technologies, UK	07923
Dulbecco’s Modified Eagle Medium, low glucose (DMEM)	Life Technologies, UK	11880
Ethanol, 200 proof	Sigma Aldrich, UK	E7023
Ethidium Homodimer	Life Technologies, UK	E1169
FastStart Universal SYBR Green Master	Roche Life Science, USA	04913850001
Foetal Bovine Serum (FBS)	Sigma Aldrich, UK	F7524
HEPES, 1M	Sigma Aldrich, UK	H0887
Hydroquinone	Sigma Aldrich, UK	H9003
Irgacure 2959	Ciba Speciality Chemicals, Switzerland	Irgacure2959
L-Glutamine, 200mM	Sigma Aldrich, UK	G7513
Methacrylic Anhydride	Sigma Aldrich, UK	27668-5
MultiScribe Transcriptase Kit (including 10x sample buffer, Deoxynucleotide triphosphates, 25x RT Random Primers)	Life Technologies, UK	4368814
Non-essential amino acids	Sigma Aldrich, UK	M7145
Paraformaldehyde	Sigma Aldrich, UK	P6148
Penicillin/Streptomycin, 10000U/10mg per mL	Sigma Aldrich, UK	P4333
Phalloidin (Alexa Fluor 488)	Life Technologies, UK	A12379
Phase Lock Gel Heavy	5 Prime GmbH, Germany	232830
Phosphate buffered saline (PBS)	Sigma Aldrich, UK	P5368
Poly(ethylene glycol) (MW3000) (PEG)	Merck Schuchardt, Germany	8.19015.1000
Primers and Probes	Life Technologies, USA	Custom order
QIAgen MiRneasy Micro Kit (including QIAzol lysis reagent and MinElute Spin Columns)	QIAgen, USA	217084
QIAshredder	QIAgen, USA	79654
RNase Free Water	Sigma Aldrich, UK	W4502
Sodium Bicarbonate	Sigma Aldrich, UK	S-8875
Triton X-100	Sigma Aldrich, UK	X100

Trypan Blue	Sigma Aldrich, UK	T8154
Trypsin-EDTA (1X concentration)	Sigma Aldrich, UK	T3924
Tyrosine-Arginine-Glycine-Aspartic acid-Serine (YRGDS)	GenScript, USA	Custom

Table S2: List of custom primers used for gene expression analysis (ordered from Life Technologies, USA).

Gene	Abbrev.	Primer Sequence		Product Size (bp)
Ribosomal protein L30	L30	**Forward**	5'-GGCAGGCGGATTCTTTACC-3'	62
		**Reverse**	5'-TAGAGGCTTTCTCTCGGACAGATG-3'	
Matrix Metalloproteinase-3	MMP-3	**Forward**	5'-TCCGCCTTTCTCAGGATGAT-3'	62
		**Reverse**	5'-GGCACCACAGGGTCATTAGG3-'	
Tissue Inhibitor of Metalloproteinase-3	TIMP-3	**Forward**	5'-AGTCTCTGTGGCCTTAAGCTTGA-3'	88
		**Reverse**	5'-TGGTCCCACCTCTCTACAAAGTTAC-3'	
Tenomodulin	TNMD	**Forward**	5'-TCTGGCGTGACGGGTCTT-3'	54
		**Reverse**	5'-AAAAAAGGCATTGAACAAAACGA-3'	
